# Hysteropexy for stage 1 uterine prolapse to prevent recurrence: a systematic review and Dutch nationwide survey

**DOI:** 10.1186/s12905-025-04174-4

**Published:** 2025-11-25

**Authors:** Mart C.P. Kortman, Claudia Manzini, Annemarie J. Goos, Hugo W.F. van Eijndhoven, Anique T.M. Grob

**Affiliations:** 1 Multimodality Medical Imaging (M3i) group, Technical Medical Centre, University of Twente, PO Box 217, Enschede, 7500 AE the Netherlands; 2 Department of gynecology), Ziekenhuisgroep Twente, Hengelo, OV the Netherlands; 3 Department of gynecology, Medisch Spectrum Twente, Enschede, the Netherlands; 4 Department of gynecology, Isala, Zwolle, the Netherlands

**Keywords:** Hysteropexy, Stage 1 uterine prolapse, Recurrence, Review, Survey

## Abstract

**Background:**

It is generally considered that women start experiencing bothersome complaints when a pelvic organ prolapse reaches stage 2. However, studies indicate that a stage 1 uterine prolapse can already cause symptoms and consensus on its treatment is lacking. The aim of this study is to identify the available literature and the clinical variance regarding concomitant surgical treatment for stage 1 uterine prolapse during surgical treatment for stage ≥ 2 anterior and/or posterior prolapse and to assess its effect on the risk of recurrence.

**Method:**

PubMed and Cochrane CENTRAL library were searched in July 2025. Studies reporting recurrence after surgical prolapse repair for stage ≥ 2 anterior and/or posterior compartment prolapse with or without concomitant hysteropexy for stage 1 uterine prolapse were included. Simultaneously, a survey regarding the clinical practice on surgical treatment of stage 1 uterine prolapse was set out to gynecologists in The Netherlands.

**Results:**

A total of 1416 records were retrieved. After screening, 16 records remained for full review. None of these studies reported the outcomes in the group of interest (i.e., stage 1 uterine prolapse). Corresponding authors were contacted for additional information on this subgroup. Three articles were included, one article performing and two not performing concomitant hysteropexy (e.g. Manchester Fothergill or Sacrospinous Hysteropexy). Reported recurrence rates were 18% in the group with concomitant hysteropexy and 20 and 60% in the groups without concomitant hysteropexy.

**Conclusion:**

This systematic review shows that literature on stage 1 uterine prolapse repair is lacking. The survey shows large clinical variance in the treatment approach of stage 1 uterine prolapse. A prospective study on the effectiveness of concomitant hysteropexy for stage 1 uterine prolapse is recommended.

**Supplementary Information:**

The online version contains supplementary material available at 10.1186/s12905-025-04174-4.

## Background

Pelvic organ prolapse (POP) is a common benign condition among ageing women. The reported incidence ranges up to 50% in parous women and there is an estimated lifetime risk of 11,1% of surgical intervention by the age of 80 [1, 2]. POP is quantified by clinical examination using the International Continence Society Pelvic Organ Prolapse Quantification (ICS POP-Q) system, published in 1996 [3]. POP-Q uses the hymen as a reference and the prolapse stage is quantified based on the distance of the anterior, posterior, and apical compartment at maximum Valsalva from the hymen.

One of the treatment options for POP is surgery. The choice of the surgical technique depends on the prolapse stage, the physician’s preference, national guidelines, and the severity of patient’s symptoms [4, 5]. Surgery is generally considered when a patient has a stage ≥ 2 prolapse, based on the ICS POP-Q guidelines, without distinction between anterior, apical or posterior compartment [3, 5]. However, studies have shown that stage 1 uterine prolapse can be bothersome [6–8]. Therefore, a stage 1 uterine prolapse could add to the prolapse complaints, when combined with anterior and/or posterior compartment prolapse.

Recent IUGA guidelines discourage the use of anatomical outcome as a measure for success [9]. However, the National Institute of Healthcare (NIH) guidelines interpret a prolapse ˂ stage 2 as an anatomical success, while more recent studies use a POP-Q C-level of −5 as anatomical success.​ [10–12] This shows that there is no consensus on the definition of anatomical success after uterine prolapse surgery. Moreover, it is unclear whether a postoperative stage 1 uterine prolapse gives a higher risk of persisting complaints and thus lowers the clinical success rate.

Apical support or level I support, as described by DeLancey, is provided by the uterosacral and cardinal ligaments [13]. Level I support failure has also been associated with a prolapse of the anterior compartment (Level II) [14, 15]. DeLancey described that, for post-hysterectomy patients, repair of level II support without the repair of level I support is prone for recurrence [13]. It is unknown whether this holds for uterine prolapse stage 1.

To summarize, it is unknown whether combined surgery for stage 1 uterine prolapse reduces POP complaints and prevents recurrence in one of the operated compartments [16]. This is the rationale of the current nationwide survey and systematic review on surgical treatment of uterine prolapse stage 1 (in this paper defined as −5 ≥ C point ≥ −2, as described by Lowder et al.) [10]. The aim is to investigate the current state of the art and clarify whether surgical repair of a stage 1 uterine prolapse at the time of a surgery for a stage ≥ 2 anterior or posterior compartment prolapse, prevents anatomical and/or symptomatic prolapse recurrence and/or recurrence surgery.

## Methods

Mesh terms and keywords related to pelvic organ prolapse, pelvic floor surgery, and recurrence were searched by the first author through PubMed and Cochrane CENTRAL library. The PubMed search strategy is reported in Table 1 (the same strategy, excluding the Mesh Terms, was used for the Cochrane CENTRAL library). The final search was conducted on July 8th 2025. There was no time restriction applied on the search, while only English sources were included. There was no restriction on uterine prolapse stage during the search. The results were exported to EndNote (Clarivate, London, UK), and duplicates were removed.

**Table 1:** PubMed search strategy

	Pelvic organ prolapse	Pelvic floor surgery	Recurrence
Mesh Term	Pelvic organ prolapse		Recurrence
Keywords	Pelvic organ prolapse(s) Cystocele Anterior vaginal wall prolapse Anterior compartment prolapse Uterus prolapse Uterine prolapse Uterine descent Descensus uteri Vault prolapse Apical prolapse Apical compartment prolapse Rectocele Enterocele Posterior vaginal wall prolapse Posterior compartment prolapse	Anterior vaginal repair Anterior colporrhaphy Posterior vaginal repair Posterior colporrhaphy Anterior colpopexy Bladder tuck Bladder lift Bladder suspension Vesicopexy Colpopexy Posterior colpopexy Rectocele repair Rectal suspension rectopexy Cystocele repair	Recurrence Recurrent Relapse Recrudescence

Studies adhering to the following criteria were included:

1.

1.1. Native tissue surgery (colporrhaphy) was performed in women with 143 symptomatic anterior or posterior POP and Hysteropexy surgery (Manchester 144 Fothergill or the Sacrospinous Hysteropexy) was performed in women with 145 stage 1, -5 ≥ C point ≥ -2, uterine prolapse (concurrent hysteropexy group).

1.2. Native tissue surgery was performed in women with symptomatic anterior or 147 posterior POP and no uterine prolapse surgery was performed in women with 148 stage 1, -5 ≥ C point ≥ -2, uterine prolapse (no hysteropexy group).

2. Statement that women with stage 1, -5 ≥ C point ≥ -2, uterine were included in 150 the study.

3. Recurrence rate was reported as outcome.

4. The amount of prolapse was graded by means of the Baden Walker or POP-Q method.

If it was not clearly stated that a subgroup of women with stage 1, −5 ≥ C point ≥ −2, uterus prolapse was included in the study, the study was excluded from our review. Study design was not a selection criterion, and studies reported only in conference abstracts were not excluded.

Eligible records were selected by screening of the title at first, then the abstract, and at last the full text. The screening was performed independently by the first and second author. Conflicts were resolved through a consensus meeting. An interlibrary request was sent when the full text paper was not available at our library. The authors of a record were contacted if it was stated that women with uterine prolapse stage 1 were included in the study, but no subgroup analysis was reported in the results or if any relevant part of the study was unclear.

A standardized data extraction form was created to retrieve the information relevant to the research question, provided in Appendix 1. The following data was extracted: reference (first author, year, journal citation), study design type, study setting, inclusion and exclusion criteria, sample size, prolapse assessment (i.e. Pelvic Organ Prolapse Quantification system or Baden-Walker), type of surgery, time to follow-up, recurrences.

Records were divided into two groups: (1) Hysteropexy surgery performed on stage 1 uterine descent (concurrent hysteropexy group) and (2) No hysteropexy surgery performed on stage 1 uterine descent. A qualitative synthesis of the results was produced for both groups (no hysteropexy group).

To assess the risk of bias of the included full-text articles, the “Let Evidence Guide Every New Decision” (LEGEND) evidence evaluation system was used [17]. The LEGEND system has evidence appraisal forms for different study designs and domains (e.g. interventional) and allows comparison of quality between different designs. The evidence appraisal forms include questions regarding validity, reliability and applicability to score the quality of the study. The included papers were assessed on quality by two authors (MCPK, CM) individually. The scores of each article were used to comment on the quality level of the body of evidence of each group. Thereby, the bias found in the articles was categorized according to the bias domains defined in the Cochrane Handbook for Systematic Reviews of Interventions [18]. 

The review was conducted in adherence to the PRISMA guidelines. The protocol of the review was registered in Prospero before implementation (CRD42023488045).

The survey was developed by three authors (MCPK, ATMG, HWFvE), all with multiple years of clinical and scientific experience in urogynecology. A request to participate was send to all members of the Dutch working party for urogynecology of the Dutch College for Obstetrics and Gynecology and distributed on LinkedIn. The responders were familiar with the guidelines surrounding surgical treatment for uterine prolapse. The survey was designed in an online environment (Microsoft Forms).

The questions addressed the clinical experience of the respondents, in which kind of hospital they work and their opinion on surgery of stage 1 uterine prolapse. The full survey can be found in Appendix 2. The results of questions two to ten were included in this paper.

## Results

**Fig. 1:**
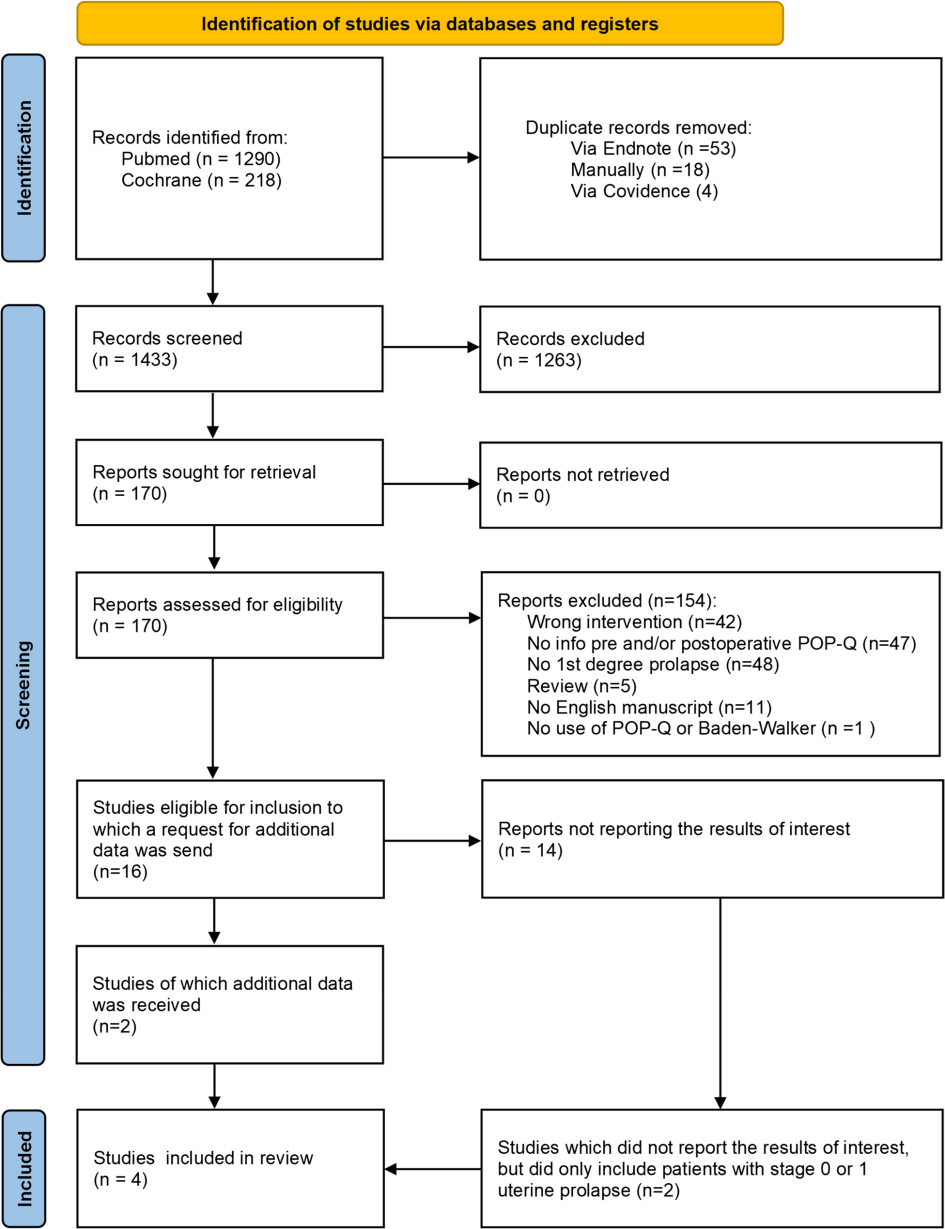
Prisma flowchart of the performed systematic review

1508 records were retrieved using the described search strategy. Of those, 75 were identified as duplicate, leaving 1433 to be screened. During title and abstract screening 1263 records were excluded and 154 were excluded during the full text screening, leaving 16 records. In these 16 records it was stated that women with stage 1 uterine prolapse were included. However, the outcomes were not separately reported for this subgroup. Therefore, a request for research data was sent to the corresponding author of each study. Two corresponding authors (Oversand et al. & Eijsink et al.) responded to our outreach [19, 20]. Resulting in two studies eligible for analysis. From the 14 which were initially discarded, two only included patients with a stage 1 or 0 uterine prolapse. It was decided to also include these papers in the analysis even if their population also included women with a stage 0 uterine prolapse [21, 22]. The PRISMA flowchart of the search can be found in Fig. 1.

An overview of the papers included in this review is shown in Appendix 1. One paper was included in group 1 (i.e., prolapse surgery performed for stage 1 uterine prolapse). Two papers were included in group 2 (i.e., no surgery performed for stage 1 uterine prolapse). One paper was included in both groups, as some of the patients with a stage 1 uterine prolapse underwent hysteropexy and others did not. The quality of the papers and the bias domains with at least a moderate change of bias are summarized in Table 2.

**Table 2:** The quality of the included studies, assessed through the LEGEND scoring method and the bias categorized according to the bias domains defined in the Cochrane handbook for systematic reviews of interventions [17, 18].

Article	Study type and quality	LEGEND quality score	Bias domains identified
Oversand et al.[20]	Longitudinal study of good quality	4 A	No bias identified
Eijsink et al.[21]	Longitudinal study of good quality	4 A	Bias in selection of participants into the study
Rudnicki et al.[23]	Randomized controlled trial of good quality	2 A	Bias due to missing outcome data
Bergman et al.[22]	Prospective cohort study of lesser quality	3 B	Bias due to missing outcome data & Bias in measurement of outcomes

The longitudinal study from 2018 performed by Oversand et al. was included in group 1 [19]. They included 153 patients scheduled for treatment of symptomatic anterior POP by means of the Manchester procedure. Patients were excluded if they previously received prolapse surgery or hysterectomy, or if an indication for a hysterectomy was present. 61 of the included patients had a stage 1 uterine prolapse. The results of the group with stage 1 uterine prolapse were not reported in the article. However, the corresponding author provided us with the number of recurrences in the apical compartment, defined as POP-Q stage >0. Eleven patients (18%) treated by means of the Manchester procedure suffered from a recurrence in the apical compartment at 1 year follow-up. The study quality was assessed through the LEGEND method as a longitudinal study, both scorers found the article to be of good quality.

The paper by Eijsink et al. included 25 patients with a stage 1 uterine prolapse [20]. The included patients at least received an anterior colporrhaphy. Of the 25 patients with a stage 1 uterine prolapse, 20 patients received concomitant hysteropexy and five patients did not. So, this paper could be included in group one and group 2. Due to the short follow-up period of 6 weeks, the researchers were unable to accurately report on recurrences. However, the symptomatic improvement was used for analysis. In the group with concomitant hysteropexy two patients (2/20) reported no improvement. In the group without concomitant hysteropexy one patient (1/5) reported no improvement. The study quality was assessed through the LEGEND method as a longitudinal study, both scorers found the study to be of good quality.

The two papers included in group 2 excluded patients with a concomitant uterine prolapse larger than stage 1. However, the study designs of the two papers differ in many aspects. The randomized controlled trial performed by Rudnicki et al. followed 78 women, who were older than 55 years, for 12 to 36 months after surgery [22]. The included patients had at least a stage 2 anterior compartment prolapse with or without a concomitant posterior prolapse, for which at least an anterior colporrhaphy was performed. Patients were ineligible to participate if they received previous pelvic or vaginal surgery, used corticosteroids, or had a history of genital or abdominal malignancies. Recurrence in any compartment (compartment specifics were not reported), defined as a POP-Q stage ≥ 2, were reported as 60.2 at 1-year follow-up and 58.8% at 3-year follow-up. Symptomatic recurrence was assessed by means of questionnaires (pelvic floor disability index), 32% of the patients reported a bulging sensation at follow-up. The study quality was assessed through the LEGEND method as a randomized controlled trial, both scorers found the article to be of good quality.

Bergman et al. included 731 women with an isolated ≥ stage 2 anterior compartment prolapse and 384 women with an isolated ≥ stage 2 posterior compartment prolapse [21] The authors did not specify whether a concomitant stage 1 apical prolapse or no apical prolapse was present. According to suture-type (rapid or slow absorption) used during surgery, they were divided into two cohorts. Follow-up was set at 12 months, and recurrence was defined as “sensation of vaginal bulging”. The authors report a 28% recurrence in the anterior colporrhaphy group and 20% recurrence in the posterior colporrhaphy group. The cause of the recurrence was not specified as no postoperative physical examination was performed. The study quality was assessed through the LEGEND method as a cohort study. After first assessment and discussion between the scorers the study was scored as a prospective cohort study of lesser quality.

A total of 36 gynecologists, of which 3 completed a fellowship urogynecology, and one gynecologist in training, with a special interest in urogynecology, responded to our request. A response rate is impossible to calculate, as the survey was shared on different platforms. The mean age of the respondents was 47 years old, with 11 years of gynecological work experience. The respondents work in a general hospital (10 (28%)), teaching hospital (23 (64%)) or in an academic hospital (3 (8%)).

The question regarding the POP-Q C-value and indication for uterine prolapse repair showed a great clinical practice variation. The responses can be found in Table 3.

**Table 3:** Responses to the question from which POP-Q C-level the respondents perform apical prolapse repair surgery

POP-Q C-level	Number of responses (%)
C −1	1 (3%)
C −2	10 (28%)
C −3	14 (38%)
C −4	9 (22%)
C −5	3 (8%)

*POP-Q* Pelvic organ prolapse quantification

Three respondents reported that they solely use supine-based POP-Q measurements as the method to establish whether uterine prolapse surgery is indicated. All other respondents either include physical examination in standing position, physical examination in the operating room, or the patient’s preference as additional method to set the indication for uterine prolapse surgery. Of the respondents, nineteen (53%) reported that they always counsel for concomitant uterine prolapse surgery, when discussing a surgery for the anterior or posterior compartment, twelve respondents occasionally discuss a concomitant procedure, and five respondents only discuss a concomitant procedure when indicated.

## Discussion

The aim of this systematic review and national survey was to identify the available literature on concomitant hysteropexy for stage 1 uterine prolapse and investigate the variation in current clinical practice in the Netherlands. Only 16 articles reported the inclusion of patients with stage 1 uterine prolapse. However, none of the articles published the specific outcomes of this subgroup. Four articles were included in the analysis. Of the four included articles, one article reported on concomitant hysteropexy surgery of stage 1 uterine prolapse, two articles reported on no concomitant hysteropexy surgery for stage 0 or 1 uterine prolapse, and one on both patients with and without concomitant surgery for stage 1 uterine prolapse. In the concomitant hysteropexy group the recurrence rate, defined as uterine prolapse >stage 0, was reported to be 18% in the apical compartment, the symptomatic improvement rate was reported to be 90% [19, 20]. In the no hysteropexy group a recurrence rate, defined as uterine prolapse >stage 1 or vaginal bulge feeling, reported as 60% and 20% respectively. In this group the symptomatic improvement rate was reported to be 75% [20–22]. The survey on stage 1 uterine prolapse surgery shows a large variation, with solely one respondent performing uterine prolapse repair from stage 2 onwards, whilst approximately 30% of the respondents operate with the cervix at POP-Q C-value − 4 or −5.

In the systematic review only four papers met the inclusion criteria. The study performed by Rudnicki et al. lacks representativeness for the entire population undergoing POP repair surgery, since they only included postmenopausal women. In clinical practice premenopausal women are treated for POP with surgical interventions, with known reported higher recurrence rates due to their younger age [23]. 

Since it has been established that the stage of prolapse and prolapse symptoms are generally poorly associated, it is important to include both objective data (e.g. anatomical data based on POP-Q) and subjective data (e.g. symptoms based on questionnaires) in the assessment of the effect of prolapse treatment [24]. The studies performed by Oversand et al. and Bergman et al. did not report on the full postoperative status of the patients [19, 21]. Oversand et al. only report POP-Q data of the apical compartment and Bergman et al. only report vaginal bulge feeling postoperatively. On the other hand, Eijsink et al. did report on the symptomatic improvement in the patients, which were similar for both the hysteropexy and non-hysteropexy groups [20]. However, the numbers are small and the follow-up period of 6 weeks very short. So no conclusions on recurrence could be drawn.

Other research has been conducted on the role of apical support in recurrence of POP. Eilber et al. performed a large-scale dataset analysis [25]. They included the data of 2756 women receiving vaginal prolapse repair surgery and assessed the 10 years reoperation rates after POP surgery with or without concomitant apical support surgery. They conclude that there is a significant difference in reoperation after solely anterior repair versus anterior repair with concomitant apical repair, favoring concomitant apical repair. This suggests a positive relation between concomitant apical repair and reduced prolapse recurrence. The study was excluded from this review since the severity (staging) of prolapse was not preoperatively specified and since there was no statement that stage 2 and higher were not included.

A relatively small but representative sample of the gynecologists with an interest in urogynecology in the Netherlands responded to our survey. The results show a great clinical variation in the indication for uterine prolapse surgery based on the POP-Q assessment. Thereby, just a few respondents only use standard physical examination to base the indication for uterine prolapse surgery as supine assessment of POP underestimates the true extent [26]. These variations in clinical practices amplify the treatment difference for patients with comparable POP. Future research should be conducted to reveal whether surgical repair of stage 1 uterine prolapse reduces anatomical and symptomatic POP recurrence, and reoperation rates. Subsequently, consensus could be reached on the treatment of uterine prolapse.

## Conclusion

This systematic review shows that literature on our research question is lacking. Therefore, an evidence based decision on performing concomitant surgery of stage 1 uterine prolapse cannot be made. According to our survey, current clinical practice shows a great variation in the treatment of stage 1 uterine prolapse. We thus conclude that a prospective study on concomitant surgical repair of stage 1 uterine prolapse is recommended.

## Supplementary Information

Supplementary Material 1.

## Data Availability

All data generated or analyzed during this study are included in this published article.

## Appendix 1– Data extraction form

*Provided through supplementary materials.*

## Appendix 2 – Questionnaire questions

**Table:**

Question	Answer options
What is your age?	
How many years of experience do you have as a gynecologist?	
In what type of department do you work?	- Academic hospital - Teaching hospital - Regional hospital
What is the title of your profession?	- Fellow urogynecology - Gynecologist - Resident obstetrics and gynecology
How to you determine the indication for uterine prolapse repair surgery?	- Physical examination (PE) in the gynecological chair - PE in upright position - PE under general anesthesia - Patient preference - Other: …
From which POP-Q C-value onward would you perform uterine prolapse repair surgery?	Choose an answer from − 11 to + 3.
What are other reasons that influence how you determine the indication for uterine prolapse repair surgery?	- Complaints of the patient - Age of the patient - Presence of cervical elongation - Level 1 evidence - Other: …
Do you always counsel the patient for a uterine prolapse repair surgery when they provided informed consent for an anterior and/or posterior prolapse repair?	- Yes - No - Sometimes - Other: …

## Abbreviations

POP: Pelvic Organ Prolapse
ICS: International Continence Society
POP-Q: Pelvic Organ Prolapse Quantification
LEGEND: Let Evidence Guide Every New Decision
